# Endocannabinoids as Markers of Sperm Quality: Hot Spots

**DOI:** 10.3389/fendo.2013.00169

**Published:** 2013-11-08

**Authors:** Mauro Maccarrone

**Affiliations:** ^1^Center of Integrated Research, Campus Bio-Medico University of Rome, Rome, Italy; ^2^European Center for Brain Research/Santa Lucia Foundation, Rome, Italy

**Keywords:** cholesterol, chromatin, epigenetic regulation, lipid rafts, receptors

Male reproductive health is under threat from a range of environmental and lifestyle assaults, including endocrine disrupters, toxic pollutants, and ionizing radiations, as well as lifestyle factors such as sexually transmitted infections, alcoholism, smoking, and anabolic steroid use. The latest potential hazard in our modern lifestyle is the use of plant-derived cannabinoids present in hashish and marijuana as recreational drugs, and more recently as therapeutic agents (1). In the last decade, a highly sophisticated endogenous cannabinoid system (ECS) has been discovered in mammals, where it regulates many physiological functions including human male reproduction (2–5). Here, I shall briefly discuss the activity of distinct ECS elements that can be useful to assess sperm function, and hence to potentially monitor sperm quality. Among others, these include the effect of type-1 cannabinoid receptor (CB_1_) in regulating energy metabolism and motility of human sperm, and that of transient receptor potential vanilloid 1 (TRPV1) channels in controlling their fertilizing ability. Remarkably, both receptors share a common natural agonist, that is the endocannabinoid (eCB) *N*-arachidonoylethanolamine (anandamide, AEA); instead, another major eCB like 2-arachidonoylglycerol (2-AG) can activate CB_1_, but is ineffective at TRPV1 receptors (6). The potential therapeutic exploitation of these ECS elements for the treatment of human infertility will be also addressed.

Human sperm express CB_1_, and its activation by AEA affects motility and acrosome reaction (AR). Both processes require energy, and a major role for glycolysis in supplying ATP for sperm motility has been recognized. Recently, human sperm exposure to methanandamide, a non-hydrolyzable analog of AEA, has been shown to significantly decrease mitochondrial transmembrane potential without triggering any mitochondria-dependent apoptotic death, and such an effect was prevented by the CB_1_ antagonist SR141716, but not by the CB_2_ antagonist SR144528, nor by the TRPV1 antagonist iodoresiniferatoxin (7). Interestingly, in the presence of glucose human sperm exposure to methanandamide for up to 18 h failed to affect sperm motility, that instead was dramatically reduced by the same substance under glycolysis blockage; again, the latter effect was prevented by SR141716 (7). Overall, CB_1_ activation induced a non-apoptotic decrease of mitochondrial potential, whose detrimental reflection on sperm motility could be revealed only when blocking glycolysis. These findings contribute to elucidate the relationship between CB_1_, energetic metabolism and mitochondria, an issue that appears relevant well beyond sperm biology. Indeed, mitochondrial CB_1_ activation has been recently reported to control energy metabolism in neurons (8), though the actual receptor localization on mitochondria remains controversial (9).

Another hot spot is the involvement of the AEA-binding TRPV1 receptor in human sperm fertilizing ability. Immunoreactivity for CB_1_ has been localized in the post-acrosomal region and in the midpiece of human sperm, whereas for TRPV1 it was restricted to the post-acrosomal region (10). Capsazepine (CPZ), a selective antagonist of TRPV1, was shown to inhibit progesterone (P)-enhanced sperm/oocyte fusion, as evaluated by the hamster egg penetration test. This inhibition was due to a reduction of the P-induced AR rate above that of spontaneous AR, which was instead increased (10). Altogether, these data demonstrate that TRPV1 plays a key-role in the human sperm fertilizing ability, by impacting on its fusion with the oocyte membrane. In line with this, a marked decrease of the ability of TRPV1 to bind its ligands has been shown in infertile versus fertile sperm, again supporting a major role for this ion channel in sperm functionality (11). On this basis, one might speculate that the reduction of AEA causes infertile sperm to lose their quiescent state and with that, the ability to prevent premature capacitation. This could then precipitate a premature AR, rendering that sperm infertile because of a reduced ability to penetrate an oocyte *in vivo*, or in assisted conception such as in *in vitro* fertilization (IVF) protocols. This hypothesis has recently found grounds through a clinical study performed on men affected by asthenozoospermia and oligoasthenoteratozoospermia (12). Indeed, AEA levels in seminal plasma were found to be halved in patients with respect to normal subjects (∼0.08 versus ∼0.20 nM). Remarkably, these differences in AEA content in men with different pathological semen subtypes were associated with poor semen quality, such as decreased sperm count and abnormal sperm motility, as well as with alterations of CB_1_ at transcriptional level (12). Therefore, evaluation of eCBs content in human sperm and/or in seminal plasma could be proposed as a novel diagnostic tool in reproductive medicine. In line with this, a marked reduction (down to ∼25%) of both AEA and 2-AG content in seminal plasma from infertile men has been recently documented (11). Instead, no significant alterations were found in sperm from infertile versus fertile men, neither for AEA nor for 2-AG (11). Collectively, these data pinpoint eCBs (and AEA in particular) as new biomarkers to determine semen quality, thus opening new avenues for the treatment of infertility in humans.

Further points of interest in the regulation of sperm quality by ECS are related to the role of membrane properties and epigenetic control of chromatin activity.

Mammalian sperm become fertile after completing capacitation, a process associated with cholesterol loss and changes in the biophysical properties of the membranes, e.g., at the level of cholesterol-rich microdomains termed lipid rafts (13). Membrane raft dynamics prepares the sperm to undergo AR, and in addition it may have a role in sperm-egg membrane interaction (14). Interestingly, CB_1_ and TRPV1 are affected by sperm membrane properties (15), and CB_1_ signal transduction in enhanced by lipid raft disruption in different neuronal and immune cells (16). In addition, the AEA congener *N*-palmitoylethanolamine (PEA), that has been shown in the male reproductive tract, modulates plasma membrane polarity with an effect on Ca^2+^ influx during the capacitation process (17). Remarkably, PEA might also affect some physiological sperm kinematic parameters (like sperm motility), thus impacting on the development of hyperactivation during capacitation, ultimately leading to idiopathic infertility (18). Taken together, further investigations into the contribution of sperm membrane lipid composition to the control of eCB signaling, and hence to its relevance for sperm quality and fertilizing ability, hold promise for a better design of preventive and/or therapeutic strategies against infertility. In this context, it remains to be assessed whether (and to what extent) sperm functionality might be affected by accumulation of AEA and congeners in intracellular stores called adiposomes (or lipid droplets), that are present in sperm (19), and are important for eCB signaling in different cell types (20, 21).

The last hot spot that I would like to address concerns chromatin remodeling and epigenetic regulation of sperm functions. Because CB_1_ activation plays a pivotal role in spermiogenesis (that is the developmental stage where DNA is remodeled), it has been recently hypothesized that regulation of the CB_1_ gene (*Cnr1*) might also influence chromatin quality in sperm (22). By using *Cnr1* null mutant (*Cnr1*^−/−^) mice, CB_1_ activation was demonstrated to regulate indeed chromatin remodeling of spermatids, *via* either increasing the levels of the *Tnp2* gene (encoding for the transition protein 2, that stimulates DNA nick repair *in vitro*), or enhancing histone displacement. Comparative analysis of wild-type, *Cnr1*^+/−^ and *Cnr1*^−/−^ animals suggested the possible occurrence of haploinsufficiency for *Tnp2* turnover under CB_1_ control, whereas histone displacement was disrupted in *Cnr1*^+/−^ and *Cnr1*^−/−^ mice to a lesser extent. Furthermore, flow cytometry analysis demonstrated that the genetic loss of *Cnr1* decreased sperm chromatin quality and was associated with sperm DNA fragmentation. Of note, this damage increased during epididymal transit, from caput to cauda (22). Collectively, these results demonstrate that the expression (and expectedly the activity) of CB_1_ controls the physiological alterations of DNA packaging during spermiogenesis and epididymal transit, which might have major implications for male fertility, given the deleterious effects of sperm DNA damage (22). On a final note, it should be recalled that the epigenetic regulation of target genes by eCBs, and conversely that of ECS genes (in particular CB_1_) by pathological conditions, are emerging as a major issue to understand the fine tuning of eCB signaling in human health and disease (23). Therefore, it can be anticipated that epigenetic studies on sperm quality and fertilizing capacity will open new avenues for preventing or curing (e.g., through a correct lifestyle) human infertility with innovative therapeutics.

In conclusion, distinct ECS elements like CB_1_ and TRPV1, along with the endogenous levels of their common ligand AEA, hold the promise to represent useful diagnostic biomarkers and therapeutic targets of male fertility defects. It seems noteworthy that, while CB_1_ has major effects also on female reproductive events (from oocyte development, to ovarian transport, and embryo implantation), apparently TRPV1 does not impact on female fertility (24), apart from generating hyperalgesia via primary sensory neurons during endometriosis (25). Therefore, the latter ion channel seems to represent an ideal target to specifically combat reproductive dysfunctions in males.
